# The effect of a positive reappraisal coping intervention and problem-solving skills training on coping strategies during waiting period of IUI treatment: An RCT

**Published:** 2017-11

**Authors:** Marzieh Ghasemi, Masoumeh Kordi, Negar Asgharipour, Habibollah Esmaeili, Maliheh Amirian

**Affiliations:** 1 *Student Research Committee, School of Nursing and Midwifery, Mashhad University of Medical Sciences, Mashhad, Iran.*; 2 *Research Center for Evidence-Based Health Care, Department of Midwifery, School of Nursing and Midwifery, Mashhad University of Medical Sciences, Mashhad, Iran.*; 3 *Research Center for Psychiatry and Behavioral Sciences, Department of Clinical Psychology, Mashhad University of Medical Sciences, Mashhad, Iran. *; 4 *Research Center for Management and Social Factors Influencing Health, Department of Biostatistics, Faculty of Public Health, Mashhad University of Medical Sciences, Mashhad, Iran.*; 5 *Department of Obstetrics and Gynecology, Faculty of Medicine, Mashhad University of Medical Sciences, Mashhad, Iran.*

**Keywords:** Problem-solving, Adaptation, Psychological, Insemination, Artificial

## Abstract

**Background:**

Waiting period of fertility treatment is stressful, therefore it is necessary to use effective coping strategies to cope with waiting period of intrauterine insemination (IUI) treatment.

**Objective:**

The aim of this study was comparing the effect of the positive reappraisal coping intervention (PRCI) with the problem-solving skills training (PSS) on the coping strategies of IUI waiting period, in infertile women referred to Milad Infertility Center in Mashhad.

**Materials and Methods:**

In this randomized clinical trial, 108 women were evaluated into three groups. The control group received the routine care, but in PRCI group, two training sessions were held and they were asked to review the coping thoughts cards and fill out the daily monitoring forms during the waiting period, and in PSS group problem-solving skill were taught during 3 sessions. The coping strategies were compared between three groups on the 10^th^ day of IUI waiting period.

**Results:**

Results showed that the mean score for problem-focused were significantly different between the control (28.54±9.70), PSS (33.71±9.31), and PRCI (30.74±10.96) (p=0.025) groups. There were significant differences between the PSS group and others groups, and mean emotion-focused were significantly different between the control (32.09±11.65), PSS (29.20±9.88), and PRCI (28.74±7.96) (p=0.036) groups. There were significant differences between the PRCI and the control group (p=0.047).

**Conclusion:**

PSS was more effective to increase problem-focused coping strategies than PRCI, therefore it is recommended that this intervention should be used in infertility treatment centers.

## Introduction

The outcomes of assisted reproductive treatments (ART) are often unpredictable and increase anxiety in patients during the waiting period (1). In a study by Ashlee and colleague the women undergoing intrauterine insemination (IUI) treatment described the waiting period as the most difficult stage of the treatment (2). The waiting period in the IUI treatment refers to the time interval between the IUI operation and the time of the pregnancy test, which is associated with severe distress in individuals (3). During the waiting period, people try to prepare themselves for receiving negative test results, so being in this period causes psychological distress in people (2).

In a study by Boivin and colleague, it was found that the use of avoidant coping strategies increased during the medical waiting period (3). It is often difficult to manage waiting period of infertility treatments. It is necessary that infertile women, who are in waiting periods, use effective coping strategies to cope with the negative emotions that surface during these periods (1).

Lazarus and Folkman specified two general forms of coping: problem-focused and emotion-focused (4). When people feel that they can do something about their problems, they are more likely to use problem-focused coping, but when they consider a problem or situation to be beyond their abilities, they use emotion-focused coping (4). Research suggests that there is a relationship between the emotion-focused coping strategy in infertile women and their general health and depression treatment (5, 6).

Most of the psychological pressures that infertile women go through are due to the use of maladaptive and ineffective coping strategies. Problem-solving is one of the most important strategies in facing infertility, its treatments, and many of the associated psychological effects (7). Problem-solving training can be defined as the process of helping a person develop the effective coping in many situations (8) and it aims to reduce and prevent psychological damage, and improve the health, which helps the person adapt to stressful life problems more effectively (9). 

So far, some studies have been conducted on the effect of problem-solving skills training on coping strategies in non-infertile people (9-11). In a study by Zanuzyan and colleague on female students at Tehran University, problem-solving skills training led to the increased use of problem-focused coping strategies (p=0.360) and seeking social support (p=0.037) (11). Despite the fact that infertility treatments are stressful, infertile couples have little desire to use psychological interventions to reduce the distress due to the infertility treatments (12).

Positive reappraisal coping intervention (PRCI) is a new intervention, designed for medical waiting periods, whose implementation does not need an in-person visit to the advisor, and moreover, the intervention is affordable (13). This technique has been used for waiting periods in IVF treatments, genetic tests, and in cases of recurrent miscarriage (14-17). A study by Lankastle and colleague (2006) showed that the use of PRCI causes a significant reduction in the use of avoidant coping strategies during the waiting period in women undergoing IVF treatment (18).

We conducted a study with the aim of comparing the effects of PRCI with the problem-solving skills training (PSS) on the coping strategies of IUI waiting period, in infertile women referred to Milad Infertility Center in Mashhad, Iran, during the years 2015 and 2016.

## Materials and methods

This three-group randomized controlled trial study was conducted on women who had been visiting Milad Infertility Treatment Center between December 2015 and June 2016, and who fulfilled the inclusion criteria for the study.

The inclusion criteria were: Iranian nationality, 18-40 yr of age, ability to read and write, primary infertility (is defined as cases in which the infertile person has not experienced pregnancy), and obtaining a score less than 28 from the General Health Questionnaire. The exclusion criteria were: suffering from a medical disease (cardiopulmonary diseases, hypothyroidism, hyperthyroidism, epilepsy, high blood pressure, and immunodeficiency diseases), smoking cigarettes or hookah, consumption of alcohol, narcotics, or stimulants, failure to complete the IUI treatment cycle, and unwillingness to continue their cooperation in the research.

The sample size was calculated based on Cohen's table, and considering a power of 80%, a confidence level of 95%, and an effect size of 70%, 33 individuals were determined to be in each group. Then, taking into account a 10% loss, 36 individuals were determined for each group (19). 

In order to prevent the dissemination of information between the groups were considered at three different times, so that after the completion of the sampling in a group, sampling was started in the other group The manner of assignment was in this way that first The names of the three groups were written on a paper and the control group was drawn for the first period, the PRCI group for the second period, and the PSS training group for the third period of time. The purposive sampling method was applied in each group.

**Measurement tools**

The coping strategy questionnaire contains 50 questions and the answers are set to a three-point Likert scale with zero meaning ‘did not use’ and three meaning ‘used a lot’. The score of the problem-focused coping strategy is obtained from the total scores of the coping strategies seeking social support, responsibility, strategic problem-solving and positive reappraisal. The score of the emotion-focused coping strategy is obtained from the total scores of face-adopted coping strategies, self-control, distancing, and avoiding escape. The minimum possible score of the problem-focused coping strategies questionnaire is zero and the maximum is 69, while the minimum possible score of the emotion-focused coping strategies questionnaire is zero and the maximum score is 81 (20). In Iran the validity of this questionnaire had been confirmed by Alipur and colleague (21). The reliability of the coping strategies questionnaire is between 0.076-0.66 through Cronbach's alpha coefficient (22) and in the present study, it was determined as a=0.086 through Cronbach's alpha coefficient too.

The General Health Questionnaire is a questionnaire containing 28 questions that measure the physical symptoms, anxiety, insomnia, social dysfunction, and severe depression. The answers are set up in a four-point Likert scale. Some of the terms are scored in reverse (the questions 1, 15-21), these questions are scored as: not at all; 3, at the usual level; 2, more than usual; 1 and far more than usual; zero, but other questions in the questionnaire are scored as: not at all; zero, at the usual level; 1, more than usual; 2 and far more than usual; 3. Minimum and the maximum acquired scores are zero and 84. The threshold score in this questionnaire is 28 and acquiring a score higher than 28 is a sign of a person's susceptibility to mental disorders (5). 

This questionnaire was completed by the participants before the intervention and obtaining a score less than 28 from the General Health Questionnaire was one of inclusion criteria for the study. The validity of the questionnaire`s Persian version had been confirmed. (20). In the study by Rasulzadeh and colleague (a=0.83) and in the present study (a=0.92) validity of the questionnaire had been confirmed by using Cronbach's alpha coefficient.

The daily monitoring form includes 46 questions related to the person's feelings, physical symptoms related to anxiety, symptoms and signs of failure and success of the treatment, a person`s assessment during the waiting period of the treatment result, and coping strategies during this waiting period. This form is a part of PRCI, which was completed each day by the PRCI group in the waiting period before getting the treatment result (18). The validity was confirmed (0.88 0.91) by Cronbach's alpha coefficient (18) and in the present study, its validity was determined (a=0.74) by Cronbach's alpha coefficient. 

The positive coping thoughts card is designed based on the positive reappraisal coping strategies and contains 10 sentences. The PRCI group repeated the sentences at least twice a day during the waiting period before getting the result of the fertility treatments. The validity of this instrument was confirmed (a=0.89) by Cronbach's alpha coefficient in the study by Lankastl and colleague. (18). 

The validity of the demographic questionnaire and information related to infertility, the daily monitoring forms, the positive coping thoughts card, and the checklist of PSS training implementation was determined by the validity of the qualitative content. After the preparation and translation under the supervision of the guidance and counsellor professors, the questionnaires were delivered to seven experts and professors of the Mashhad University of Medical Sciences for assessment. The final tool was used after considering the suggestions and necessary revisions.

**Intervention**

The infertile women who visited the center in order to plan for the IUI treatment and/or to undergo a basic ultrasonography (during days 2-3 of the menstrual cycle) on the first day of treatment and who fulfilled the inclusion criteria for the study were recruited for this study. The intervention was done by the researcher. The duration of each session was set to 45-50 min according to the time when the subjects presented themselves at treatment centers.

The problem-solving skills, according to Dezorila and Goldfrid method (8), were taught in the three individual sessions (Table I). 

In the PRCI group, two individual sessions were held (Table II) and the participants were asked to repeat the positive coping thoughts at least twice a day while waiting for the treatment result (1, 18, 24). 

The control group members received the center’s routine care, and presented themselves at Milad Infertility Treatment Center on days 2-3, 9-12, and 14-15 of the menstrual cycle to undergo an ultrasonography and determine the remedial measures for the IUI treatment, and finally, the coping strategies were compared between the three groups on the 10^th^ day of IUI waiting period.

**Ethical consideration**

After the approval of the research subject by the ethics committee of Mashhad University of Medical Sciences (IR.MUMS REC.1394.531) and after obtaining the consent of the officials of Milad Infertility Treatment Center.

**Statistical analysis**

After collection and coding, the data was entered into the computer, and data analysis was carried out using the Statistical Package for the Social Sciences (SPSS, version 16, SPSS Inc, Chicago, Illinois, USA). To describe the characteristics of the subjects in each of the three groups, descriptive statistics were used, including the statistical indicators like mean, standard deviation, and frequency distribution tables. The Chi-square test was used in order to investigate the homogeneity of the qualitative variables in the three groups and analysis of covariance for that of the quantitative variables. The p-value<0.05 were considered as the significance level.

## Results

**Figure 1:**
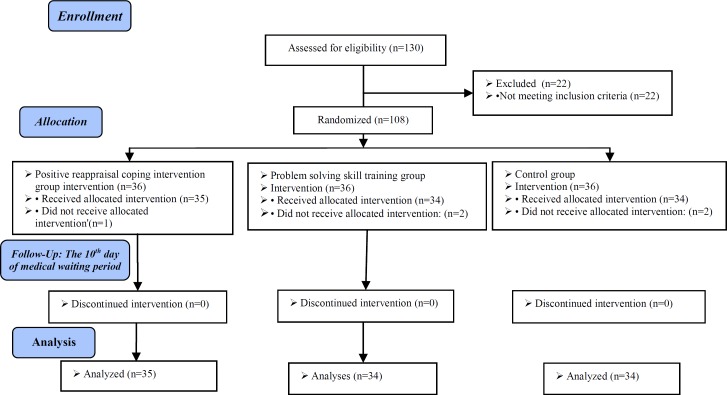
Describing the stages of performing the intervention

At the beginning of the study, 108 individuals were included in the research, out of whom two individuals from the control group and one from the PRCI group were excluded from the study due to the cancellation of their treatment cycles, and from the PSS training group, one person was excluded due to the cancellation of her treatment cycle and one due to her unwillingness to continue participating in the research (Figure 1).

The subjects were homogeneous in terms of level of education (p=0.853), family income (p=0.855), cause of infertility (p=0.824), and experience of using assisted reproductive treatments (p=0.410) (Table III).

The three groups were homogeneous in terms of the variables of general health scores (p=0.712) and age (p=0.396) (Table IV).

The median and interquartile range for the time of planning their fertility in the control group was 3 and 3.0 respectively, in the PSS training group was 3.5 and 3.0, respectively, and in the PRCI group were 3 and 2, respectively, and the three groups were homogeneous in terms of this variable (p=0.651).

The ANCOVA (Analyze of Covariance) test was used for the controlled mean scores of the coping strategies before the intervention. The results showed that on the 10th day of the waiting period of IUI treatments, there were significant differences between the three groups in the problem-focused coping strategies (p=0.025). The mean of the problem-focused coping strategy increased in the PSS training group, but it decreased in the two other groups (Table V). Comparisons pair wise between the groups showed that there were significant differences between the PSS training group with the control group (p=0.010), and there were significant differences between the PSS training group with the PRCI (p=0.047).

There were significant differences between the three groups in the emotion-focused coping strategies (p=0.036) too. The mean of the emotion-focused coping strategy increased in the control group, but it decreased in the two other groups (Table V). A significant difference was found between the control group and the PRCI group (p=0.047).

On the 10^th^ day of the medical waiting period of the IUI treatments, there were significant differences between the three groups in terms of the social support and planned problem-solving coping strategies among the components of the problem-focused coping strategy, and the avoidant coping strategy among the components of the emotion-focused coping strategy.

There were significant differences between the three groups in terms of social support (p=0.004). The mean of the seeking social support coping strategy increased in the PSS training and PRCI groups but decreased in the control group (Table VI). A paired comparison between the groups showed that there was a significant difference between the control group and the PSS training group (p=0.001). 

There were significant differences between the three groups in planned problem-solving coping strategies (p=0.003); the mean of the planned problem-solving coping strategy increased in the PSS training group, whilst it decreased in the other two groups (Table VI). A paired comparison between the groups showed that there were significant differences between the problem-solving skills training group with the control group (p=0.003) on the one hand and the PRCI group (p=0.003) on the other hand. 

There were significant differences between the three groups in terms of the avoidant coping strategy (p=0.006). The mean of the avoidant coping strategy increased in the control group whilst it decreased in the other two groups (Table VI). 

A paired comparison between the groups showed that there were significant differences between the control group and the problem-solving skills training group (p=0.002) and between the control group and the PRCI group (p=0.038).

**Table I:** Topics session of problem-solving skills training group

**Topics of each session**	**Session**
Information's were given about the research objectives, infertility and IUI treatment. Problem solving steps and the correct approach to solving the problem were discussed. The participants were asked to write a list of problems that people have during the course of treatment with IUI and determine the most important issue.	First session Days 2-3 of the menstrual cycle
The brainstorming method to provide solutions for their problems were taught. The participants were asked to determine a list of solutions for solving their problems.	Second session Days 9-12 of the menstrual cycle
The advantages and disadvantages of the implementation of each solution were discussed. The evaluation of the effectiveness of the solutions, and the strategy of returning to the previous level in case of ineffective results of the proposed solution were taught. They were asked to implement the PSS training in their daily lives during the waiting period of the treatment and to record the implementation in the PSS training checklist.	Tthird session Days 14-15 of the menstrual cycle
The participants were asked to call the researcher in the case they had problems with completing the checklist of PSS implementation.	Two phone sessions Days 2 and 7 of waiting period

IUI: Intrauterine insemination

**Table II:** Topics session of positive reappraisal coping intervention group

**Topics of each session**	**Session**
Information's were given about the research objectives, infertility and IUI treatment. The variety of coping strategies and positive reappraisal coping strategies were discussed.	First session Days 2-3 of the menstrual cycle
10 positive coping thoughts card were explained by an example. The way of completing the daily monitoring form was explained The participants were asked to repeat the positive coping thoughts at least twice a day while waiting for the treatment result	Second session Days 9-12 of the menstrual cycle

IUI: Intrauterine insemination

**Table III:** Frequency distribution of infertile women undergoing assisted reproductive treatments based on level of education, socioeconomic status, family income, cause of infertility, and prior use of assisted reproductive treatments in the three groups: control, problem-solving skills training, and positive reappraisal coping intervention

**Variable**		**Group**			
		**Control**	**Problem solving skills training**	**Positive reappraisal coping intervention**	**Test results**
The woman's level of education					
	Elementary school Middle school High school University	7 (19.4) 5 (13.9) 8 (22.2) 16 (44.4)	4 (11.1) 6 (16.7) 11 (30.6) 15 (41.7)	2 (5.6) 4 (11.1) 16 (44.4) 14 (38.9)	X^2^= 0.31 df= 2 p= 0.853^a^
Family income					
	Lower than enough Almost enough More than enough	1 (27.8) 25 (69.4) 1 (2.8)	12 (33.3) 23 (63.9) 1 (2.8)	1 (27.8) 25 (69.4) 1 (2.8)	X^2^= 0.31 df= 2 p= 0.855^a^
Cause of infertility					
	Male factor Female factor Joint factor Unknown factor	4 (11.1) 10 (27.8) 9 (25) 13 (36.1)	4 (11.4) 14 (40) 7 (20) 10 (28.6)	4 (16.7) 15 (41.7) 7 (19.4) 8 (22.2)	ExactX^2^= 3.01 p= 0.824^b^
Prior use of assisted reproductive techniques					
	None IUI IVF Other	17 (47.2) 14 (38.9) 0.00 5 (13.9)	20 (55.6) 9 (25) 0.00 7 (19.4)	19 (52.7) 13 (36.1) 1 (2.8) 3 (8.4)	ExactX^2^= 0.39 p= 0.410^b^

All data is presented as n(%)

IUI: Intrauterine insemination

IVF: In vitro fertilization

a . Kruskal-Wallis

b . Fisher exact

**Table IV:** Comparing the mean and standard deviation of the subjects' age and general health before the intervention in the three groups: control, problem-solving skills training, and positive reappraisal coping intervention

**Variable**	**Group**			**Test results**
	**Control**	**Problem-solving skills training**	**Positive reappraisal coping intervention**	
General health	25.85 ± 3.39	25.28 ± 4.20	26.15 ± 3.70	X^2^= 0.68 P= 0.712 a
Age	27.77 ± 4.8	28.41 ± 4.22	29.22 ± 4.40	F= 0.935 p= 0.396^b^

All data is presented as Mean ± SD

a . Kruskal-Wallis

b . One-way ANOVA

**Table V:** Comparing the mean and standard deviation of the emotion-focused coping strategy and problem-focused coping strategy before the intervention and on the tenth day of the medical waiting period of assisted reproductive treatments in the three groups: control, problem-solving skills training, and positive reappraisal coping intervention

**The variable coping strategy**		**Group**			**Result of Ancova test**	
		**Control**	**Problem-solving skills training**	**Positive reappraisal coping intervention**	**Group effect**	**Covariate effect**
Problem-focused						
	Before intervention Waiting period Effect size	29.11 ± 9.067 28.54 ± 9.70 -	29.37 ± 10.47 33.71 ± 9.31 0.004	31.13 ± 10.43 30.74 ± 10.96 0.066	F= 3.81 p= 0.025^a^	F= 37.28 p< 0.001
Emotion-focused						
	Before intervention Waiting period Effect size	30.02 ± 9.91 32.09 ± 11.65 -	32.55 ± 6.97 29.20 ± 9.88 0.062	33.80 ± 11.64 28.74 ± 7.96 0.033	F= 3.43 p= 0.036^b^	F= 29.68 p< 0.001

All data presented as Mean ± SD.

a . Comparisons pairwise between groups showed, significant differences between the problem-solving skills training with control (p=0.010) and positive reappraisal coping intervention group (p=0.047)

b . Significant difference between positive reappraisal coping intervention the control group

**Table VI:** Comparing the mean and standard deviation of the coping skills: seeking social support, accepting responsibility, planned problem solving, positive reappraisal, confrontive coping, self-control, distancing, and avoidance, before the intervention and on the tenth day of the medical waiting period of assisted reproductive treatments in the three groups: control, problem solving skills training, and positive reappraisal coping intervention

**Variable Coping strategy**		**Group**			**Results of ANCOVA**	
		**Control**	**Problem-solving skills training**	**Positive reappraisal coping intervention**	**Group effect**	**Covariate effect**
Seeking social support						
	Before intervention Waiting period Effect size	8.83 ± 3.60 8.32 ± 3.20 -	8.08 ± 4.10 10.61 ± 4.57 0.023	8.02 ± 3.90 9.05 ± 4.92 0.105	F = 5.81 p = 0.004^a^	F = 53.02 p < 0.001
Accepting responsibility						
	Before intervention Waiting period Effect size	4.5 ± 2.33 4.23 ± 2.00 -	5.10 ± 1.97 3.55 ± 2.01 0.009	5.02 ± 2.41 3.85 ± 1.90 0.021	F = 1.108 p = 0.334	F = 4.125 p = 0.045
Planned problem solving						
	Before intervention Waiting period Effect size	6.05 ± 3.33 6.40 ± 3.04 -	5.9 ± 3.40 7.55 ± 3.34 <0.001	7.47 ± 3.23 6.20 ± 3.07 0.085	F = 6.227 p = 0.003^b^	F = 24.502 p < 0.001
Positive reappraisal						
	Before intervention Waiting period Effect size	9.41 ± 4.05 10.14 ± 3.84 -	10.52 ± 4.20 11.82 ± 3.38 0.019	10.61 ± 3.61 11.62 ± 4.02 0.037	F = 1.995 p = 0.141	F = 22.130 p < 0.001
Confrontive coping						
	Before intervention Waiting period Effect size	5.50 ± 2.21 5.20 ± 2.58 -	6.22 ± 2.40 5.58 ± 3.12 0.011	6.11 ± 2.88 4.8 ± 2.5 0.001	F = 0.984 p = 0.378	F = 18.96 p < 0.001
Self-control						
	Before intervention Waiting period Effect size	8.38 ± 3.7 8.91 ± 4.87 -	8.27 ± 2.94 9.05 ± 3.00 0.021	10.27 ± 6.63 8.20 ± 3.44 0.001	F = 1.849 p = 0.141	F = 22.130 p < 0.001
Distancing						
	Before intervention Waiting period Effect size	7.16 ± 3.22 8.41 ± 3.81 -	7.97 ± 2.65 6.50 ± 3.03 0.016	7.77 ± 3.19 7.05 ± 6.30 0.024	F = 1.36 p = 0.259	F = 6.657 p = 0.011
Avoidance						
	Before intervention Waiting period Effect size	8.75 ± 3.86 9.91 ± 3.94 -	9.86 ± 3.57 7.61 ± 3.86 0.043	9.63 ± 3.24 8.62 ± 2.96 0.095	F = 5.320 p = 0.006^c^	F = 25.22 p < 0.001

Data presented as Mean ± SD

a . Significant difference between problem-solving skills training and the control group.

b . Significant differences between the problem-solving skills training with control and positive reappraisal coping intervention group

c . Significant differences between the control group with problem-solving skills training and positive reappraisal coping intervention group

## Discussion

In this study, the problem-focused coping strategies were found to have increased in the PSS training group. In the study by Goorhan and colleague, after two sessions of counseling for problem-focused coping strategies among the women undergoing the treatment, there was no statistically significant difference between the intervention and the control groups (25). In that study, one consultation session was held as a group session and one consultation session was held with each individual. The exclusion criteria were not being under psychiatric treatment and the lack of psychiatric disorders during the study. However, in the present study, PSS training was given individually during three sessions. Also, the general health score above 28 was the exclusion criteria, which can affect the study results. In the study by Heidari and colleague, there was a relation between general health and coping strategies in infertile women. Infertile people who used problem-focused coping strategies had attained higher general health in comparison to the infertile people who used less of the problem-focused coping strategies (6). 

In our study, the coping strategies of thoughtful problem-solving had increased in the PSS training group. In the study of Zanuzyan and colleague, after seven sessions of PSS training, the mean scores of coping strategies of thoughtful problem-solving in the PSS training group were significantly increased compared to the control group (11). This is aligned with the result of the current study about the effect of the PSS training on problem-focused coping strategies. Also, in the study by Chynaveh and colleague, after six sessions of problem-solving skill training, the students’ coping strategies in the intervention group were significantly different from the control group which is aligned with the result of the current study about the effect of PSS training on changing problem-focused coping strategies (26). The PSS training focuses on the training of the skills and problem-solving attitude and increases a person’s skill in recognition of the problem and different solutions for it (27). 

In our study, there is a significant difference between emotion-focused coping strategies in the PRCI group and the control group. In the study by Lee and colleague, crisis intervention was conducted by nurses at an infertility center, after three sessions of intervention. The emotion-focused coping strategy in the waiting period for the treatment result with IVF had no significant difference in the intervention and the control groups (28), which is not aligned with the result of the current study. The present study noted significant differences in the emotion-focused coping strategies in the PRCI group and the control group. In the study by Lee and colleague, the used intervention includes a video training session of IVF treatment levels, a session about muscle relaxation training, and a session of counseling based on the cognitive behavioral approach to IVF treatment. In the current study, in the PRCI group, two sessions of training in a variety of coping strategies included the positive reappraisal coping strategies and the way of using a positive coping thoughts card and filling out the daily monitoring form during the waiting period. Positive reappraisal coping intervention is an intervention that has been designed for the waiting period of the result of assisted reproductive therapy. The idea of this intervention is to create positive thinking with a cognitive process (15). Positive reappraisal coping intervention is designed based on the positive reappraisal coping strategies (23). Such positive reappraisal coping strategies can help people cope with unpredictable and long periods of time like the waiting period. Positive reappraisal coping strategies lead to a reassessment of a situation and the emphasis is on discovering the advantages and the positive aspects in stressful situations (1, 24). 

In our study, emotion-focused coping strategies increased in the control group and reduced in the other two groups. In some studies, there is a relation between the emotion-focused coping strategies in infertile women and their general health and depression (18, 19). It is possible that emotion-focused coping strategies have negative effects in stressful and crises situations, where people experience even more excitement (5). Since the result of pregnancy test is unchangeable and uncontrollable, it is difficult to cope with the waiting period. Being in the waiting period, cause psychological responses, stress and anxiety and increasing the use of emotion-focused coping strategies in the people. Since the result of a pregnancy test is unchangeable and uncontrollable, it is difficult to cope with the waiting period. Being in the waiting period may cause psychological responses, stress, and anxiety, and increase the use of emotion-focused coping strategies in people (18). In the present study, the avoidant coping strategy increased in the control group, and decreased in the PRCI and the PSS training groups. In a study by Lankastle and colleague, during the waiting period of IVF treatment, the avoidant coping strategy decreased in the intervention group and increased in the control group, which is consistent with the results of the present study (18). In a study by Jaffe and colleague, there was a relationship between the use of avoidant coping strategy and increased distress in infertile women. Long-term use of the avoidant coping strategy prevents psychological compromise and increases distress symptoms such as depression (29).

Among the strengths of this study, we can refer to performing interventions for the waiting period during fertility treatments, as well as the adaptability of the training sessions to the therapeutic program intended for the subjects. However, among the weaknesses of the study, we can refer to the fact that the study is not double-blinded, which reduces the ability of the study to be generalized.

## Conclusion

According to the impact of problem-solving skills training observed on the problem-focused coping strategies, it is suggested that this intervention should be included in the care plans of infertility treatment centers so that infertile women can be helped to cope with the medical waiting period of assisted reproductive treatments.

## Note

***Registration ID in IRCT: IRCT2016020926490N1***

***This article extracted from M.Sc. Thesis. (Marzieh Ghasemi)***
